# *Clostridium* strain FAM25158, a unique endospore-forming bacterium related to *Clostridium tyrobutyricum* and isolated from Emmental cheese shows low tolerance to salt

**DOI:** 10.3389/fmicb.2024.1353321

**Published:** 2024-02-13

**Authors:** Lucija Prinčič, Johanna Burtscher, Paul Sacken, Tina Krajnc, Konrad J. Domig

**Affiliations:** ^1^Department of Food Science and Technology, Institute of Food Science, University of Natural Resources and Life Sciences, Vienna, Vienna, Austria; ^2^Biotechnical Faculty, University of Ljubljana, Ljubljana, Slovenia

**Keywords:** *Clostridium tyrobutyricum*, cheese quality, late blowing, characterization, sucrose

## Abstract

The genus *Clostridium* is a large and diverse group of species that can cause food spoilage, including late blowing defect (LBD) in cheese. In this study, we investigated the taxonomic status of strain FAM25158 isolated from Emmental cheese with LBD using a polyphasic taxonomic and comparative genomic approach. A 16S rRNA gene sequence phylogeny suggested affiliation to the *Clostridium sensu stricto* cluster, with *Clostridium tyrobutyricum* DSM 2637*^T^* being the closest related type strain (99.16% sequence similarity). Average Nucleotide Identity (ANI) analysis revealed that strain FAM25158 is at the species threshold with *C. tyrobutyricum*, with ANI values ranging from 94.70 to 95.26%, while the digital DNA-DNA hybridization values were below the recommended threshold, suggesting that FAM25158 is significantly different from *C. tyrobutyricum* at the genomic level. Moreover, comparative genomic analysis between FAM25158 and its four closest *C. tyrobutyricum* relatives revealed a diversity of metabolic pathways, with FAM25158 differing from other *C. tyrobutyricum* strains by the presence of genes such as *scrA*, *srcB*, and *scrK*, responsible for sucrose utilization, and the absence of many important functional genes associated with cold and osmolality adaptation, which was further supported by phenotypic analyses. Surprisingly, strain FAM25158 exhibited unique physiologic traits, such as an optimal growth temperature of 30°C, in contrast to its closest relatives, *C. tyrobutyricum* species with an optimal growth temperature of 37°C. Additionally, the growth of FAM25158 was inhibited at NaCl concentrations higher than 0.5%, a remarkable observation considering its origin from cheese. While the results of this study provide novel information on the genetic content of strain FAM25158, the relationship between its genetic content and the observed phenotype remains a topic requiring further investigation.

## 1 Introduction

Bacteria of the genus *Clostridium* are Gram-positive, anaerobic, spore-forming, rod-shaped organisms belonging to the phylum *Bacillota*. The genus *Clostridium* was first described in 1880 with the type species *Clostridium butyricum* (Prażmowski, 1880). For over a century, all anaerobic spore-forming bacteria were assigned to the genus *Clostridium*; consequently, this genus included a large number of species with great phylogenetic and phenotypic diversity (Lawson and Rainey, 2016). In recent years, the understanding of *Clostridium* taxonomy has been improved by complementing the phenotypic characterization with genome-based taxonomic approaches, resulting in the reclassification of numerous *Clostridium* species into a number of different orders, families, and genera (Yutin and Galperin, 2013; Lawson and Rainey, 2016; Cruz-Morales et al., 2019). It is now generally accepted that *Clostridium* Cluster I, or *Clostridium sensu stricto* comprises the “true” representatives of the genus (Collins et al., 1994).

To date, *Clostridium sensu stricto* encompass 118 species with validly published names (LPSN).^1^ Some species include well-known human and animal pathogens such as *Clostridium botulinum* and *Clostridium tetani*, as well as non-pathogenic species such as *Clostridium thermocellum*, *Clostridium acetobutylicum*, and *Clostridium beijerinckii* useful in industrial biotechnology (Paye et al., 2016; Du et al., 2020; Vieira et al., 2020). Many of the species play a role in the microbial spoilage of food, particularly dairy products. In this context, butyric acid bacteria (BAB) are the most relevant for the dairy industry (Doyle et al., 2015). BAB are capable to convert lactate into butyrate, acetate, H_2_, and CO_2_, resulting in a type of cheese spoilage called “late blowing” defect (LBD) (Brändle et al., 2016). This spoilage is characterized by the formation of slits, cracks, and irregular eyes, as well as pronounced off-flavors in hard and semi-hard cheeses. Although *Clostridium* species *butyricum*, *sporogenes*, and *beijerinckii* have been associated with LBD (Klijn et al., 1994; Klijn et al., 1995; Cocolin et al., 2004; Le Bourhis et al., 2007; Garde et al., 2011a; Brändle et al., 2018b), it is known that *Clostridium tyrobutyricum* is the main cause of this defect in most cheese types. However, a remarkable degree of phenotypic and genetic diversity has been widely reported among strains of the *C. tyrobutyricum* species (Garde et al., 2012; Ruusunen et al., 2012; Bermudez et al., 2016; Burtscher et al., 2020; Podrzaj et al., 2020; Podrzaj et al., 2022).

While exploring the diversity of *C. tyrobutyricum* strains, a strain designated as Cl_52 (named FAM25158 in this study) showed unique phenotypic characteristics as it lacked the ability to germinate and produce gas and organic acids in a cheese-like medium (Podrzaj et al., 2020). In addition, fingerprinting studies demonstrated low similarity of strain FAM25158 to the fingerprints of other *C. tyrobutyricum* strains (Burtscher et al., 2020). Furthermore, genomic comparison of *C. tyrobutyricum* strains revealed a distant genetic relationship between strain FAM25158 and 28 *C. tyrobutyricum* strains (Podrzaj et al., 2022). Therefore, in this study, we aimed to extend the investigation of strain FAM25158 to elucidate its characteristics and assess its significance. Our specific objectives were to characterize the morphology, physiology, and chemotaxonomy of strain FAM25158, and to perform a comparative analysis with closely related *C. tyrobutyricum* strains. In addition, we conducted a comparative genomic analysis between FAM25158 and closely related *Clostridium* strains to identify strain FAM25158 down to the species level and to detect genetic elements putatively involved in the phenotype of this strain.

## 2 Materials and methods

### 2.1 Bacterial strains and culture conditions

Five strains, including *Clostridium* sp. FAM25158 (synonym Cl_52), *C. tyrobutyricum* Cl_64, *C. tyrobutyricum* Cl_117, *C. tyrobutyricum* Cl_238, and *C. tyrobutyricum* DSM 2637*^T^* were used in this study. The strain FAM25158 was originally isolated in the 1960s from Emmental cheese and was provided by Agroscope, Switzerland. The type strain *C. tyrobutyricum* DSM 2637*^T^* was obtained from the German Collection of Microorganisms and Cell Cultures GmbH, and was used as a reference strain. Strains Cl_64 and Cl_117 were isolated from raw milk, while strain Cl_238 was isolated from hard cheese, as described in previous publications (Brändle et al., 2018a,b). These strains were selected as they represented the closest relatives of strain FAM25158 based on (i) MALDI-TOF MS spectrum similarity, (ii) whole-genome based phylogeny, and (iii)16S rRNA gene sequence similarity, respectively (Podrzaj et al., 2020; Podrzaj et al., 2022). Throughout this study, *C. tyrobutyricum* strains Cl_64, Cl_117, Cl_238, and DSM 2637*^T^* were used as reference strains.

Strains were maintained at −80°C in Reinforced Clostridial Medium (RCM, Merck, Germany) with 20% (w/v) glycerol. Each strain was revived by streaking from frozen stock onto RCM agar and subsequent incubation at 37°C for 48–72 h under anaerobic conditions. The anaerobic environment was maintained using a jar gassing system and a gas mixture containing 80% N_2_, 10% CO_2_, and 10% H_2_ (Don Whitley Scientific, West Yorkshire, UK). These anaerobic conditions remained consistent throughout all experimental procedures unless otherwise specified.

### 2.2 Morphological, physiological, and biochemical characterization

The morphological characteristics of the strains tested were investigated using cells cultivated anaerobically on RCM agar at 37°C for 48–72 h. Cell morphology was observed under phase contrast microscopy using an Olympus BX41 microscope (Olympus, Tokyo, Japan) with a phase-contrast objective. Magnification was accomplished with a 100 × oil immersion objective and 10 × ocular magnification, resulting in a total magnification of 1,000 × . Gram-staining of cells was carried out using Hucker’s modification method (Hucker, 1921). Spores were produced according to a protocol previously described by Podrzaj et al. (2020). Endospore formation was determined by staining the spores with crystal violet and safranin. Cell motility was determined by observing the growth spread of cells in test tubes containing semi-solid (0.3% agar, w/v) RCM medium after incubation at 37°C for 3 days and confirmed microscopically by assessing motility in a hanging-drop preparation. Catalase activity was determined by the production of O_2_ using 3.0% (v/v) H_2_O_2_. Oxidase activity was determined using 1.0% (v/v) p-tetramethyl phenylenediamine solution (bioMérieux, France).

To determine the growth range and optimum growth conditions, the growth of the strains tested was investigated at various temperatures, salinities, and pH values. The temperature range for growth was examined in RCM broth at 4, 10, 14, 20, 21, 22, 25, 27, 30, 32, 37, 40, 42, and 45°C. The pH range for growth was determined using RCM adjusted to pH values ranging from 3 to 11 (with 0.5 pH unit increments). The pH was adjusted using 1 N NaOH or 1 N HCl. Salt tolerance was tested in RCM broth supplemented with NaCl concentrations ranging from 0.5 to 8.0% (w/v). Two separate biological replicates were performed and each replicate included inoculation of three technical broth replicates. For each analysis, a single colony of each strain grown on RCM agar was inoculated into 5 mL of RCM broth and incubated at 37°C for 20–24 h under anaerobic conditions. These overnight cultures were then used to inoculate 5 mL of the appropriate RCM broth at a starting concentration of approximately 2–3 log_10_ CFU/mL of RCM broth, determined by adjusting and measuring the optical density at 600 nm (OD_600_) to ∼0.05. To determine the initial population of inoculated RCM broth, all inoculated RCM broth samples were plated in technical duplicates onto RCM agar. Colonies were enumerated after 48–72 h of anaerobic incubation at 37°C. Tubes were incubated at the specific test temperatures or at 30 and 37°C for the NaCl concentration and pH tests. Growth was evaluated after appropriate time intervals by measuring the OD_600_. Results were expressed as the difference between the mean OD_600_ at the end of the incubation period (48 h) and the mean OD_600_ at the beginning of the incubation period (0 h), unless otherwise specified.

The fermentation/oxidation profile, and substrate utilization as sole carbon and energy sources and enzymatic profiles were determined using the API 20A and API-ZYM kits (bioMérieux) at 37°C for 48 h according to the manufacturer’s instructions.

### 2.3 Chemotaxonomic characterization

Cellular fatty acid and polar lipid analyses of strain FAM25158 and the four reference *C. tyrobutyricum* strains were carried out by DSMZ Services, Leibniz-Institut DSMZ—Deutsche Sammlung von Mikroorganismen und Zellkulturen GmbH, Braunschweig, Germany. For cellular fatty acids, the strains were grown anaerobically on RCM agar at 37°C. Cellular fatty acid methyl esters (FAMEs) were obtained from the cells by saponification, methylation, and extraction according to the protocol described by Sasser (1990). Cellular FAMEs were then separated by gas chromatography (GC) and detected by a flame ionization detector using the Sherlock Microbial Identification Systems (MIS; MIDI; Microbial ID). The analysis was supplemented by a GC-MS run on an Agilent GC-MS 7000D system for identity confirmation. Peaks were identified based on retention time and mass spectra. Polar lipids were extracted from overnight cultures in RCM broth incubated at 37°C using a chloroform-methanol: 0.3% aqueous NaCl mixture, and recovered in the chloroform phase according to a modified procedure of Blight and Dyer (1959). Polar lipids were separated by two dimensional silica gel thin layer chromatography (TLC) using chloroform:methanol:water in the first dimension and chloroform:methanol:acetic acid:water in the second dimension. Total lipid material was detected using molybdatophosphoric acid and specific functional groups were detected using spray reagents specific for defined functional groups (Tindall et al., 2014).

### 2.4 Phylogenetic analyses based on the 16S rRNA gene sequence analysis

Phylogenetic analysis between strain FAM25158 and closely related *Clostridium* species was performed using 16S rRNA gene sequences. The 16S rRNA gene sequence of strain FAM25158 was obtained from the National Center for Biotechnology Information (NCBI) GenBank database^2^ and used to query the EzBioCloud database^3^ and BLAST program^4^ to identify its closest relatives. Phylogenetic analysis was further carried out by comparing the query sequence with closely related type strains (18 type strains with identity higher than 93%) from the *Clostridium sensu stricto* cluster and the type strain of the *Clostridium sensu stricto* cluster *C. butyricum* ATCC 19398*^T^* (Supplementary Table 1). The 16S rRNA gene sequences were aligned using the ClustalW tool (Thompson et al., 1994) within the MEGAX v10.2.6 software (Kumar et al., 2018). The Maximum-Likelihood (ML) method using the Kimura two-parameter substitution model (Kimura, 1980) with 1,000 bootstrap replicates was used to reconstruct a phylogenetic tree. The 16S rRNA gene sequence of *Clostridium proteolyticum* DSM 3090*^T^* (a representative member of *Clostridium* cluster II) was selected as an outgroup. The 16S rRNA gene sequences of *Clostridium sensu stricto* species were obtained from whole genomes, where available, or retrieved directly from the NCBI RefSeq database.^5^

### 2.5 Comparative genomics and phylogenomic analyses

Genomic sequences of FAM25158 and all available genomes of all *Clostridium* species were retrieved from the NCBI GenBank database on August 8, 2023. Average Nucleotide Identity (ANI) values between FAM25158 and the 2,768 *Clostridium* genomes were determined using the python script PyANI v0.2.7^6^ which estimates ANI based on BLAST+ (ANIb) (Camacho et al., 2009). Based on the results of the ANI analysis, the 74 *Clostridium* genomes, that showed >75% ANI to strain FAM25158, were selected to calculate the digital DNA-DNA hybridization (dDDH) values using the Genome Blast Distance Phylogeny (GBDP) algorithm via the Genome-to-Genome Distance Calculator (GGDC)^7^ by employing BLAST+ alignment and the recommended formula 2 (optimized for draft genome sequences) (Meier-Kolthoff et al., 2013). The obtained sets of intergenomic distances were converted into a distance matrix and imported into MEGAX software to build a Neighbor-Joining (NJ) phylogenomic tree. Additionally, the genome sequence of FAM25158 was submitted to the Type (Strain) Genome Server (TYGS)^8^ platform for a whole genome-based taxonomic analysis using default parameters as described elsewhere (Liu et al., 2015).

The basic features of the genomic profile of strain FAM25158 were compared with *C. tyrobutyricum* Cl_64, *C. tyrobutyricum* Cl_117, *C. tyrobutyricum* Cl_238, and *C. tyrobutyricum* DSM 2637*^T^*. First, the protein-coding genes were predicted and re-annotated using Prokka suite v1.14.6 as described previously (Podrzaj et al., 2022). Then, InterProScan v5.44-79.0 (Jones et al., 2014) was used to obtain additional annotations for the genes annotated by Prokka as encoding hypothetical proteins. The pangenome of FAM25158 and four reference *C. tyrobutyricum* strains was constructed using the Prokka resultant GGF files as an input, and the families of homologous genes for the five strains were computed using Roary (Page et al., 2015) with minimum identity cutoff of 95%. Clusters of homologous genes were defined as core (conserved for all strains), accessory (shared by more than two strains), and unique (strain-specific) genes. Functional assignments of core and unique genes were performed based on similarity searches against the Clusters of Orthologous Groups (COGs) using eggNOG-mapper v2.1.9 (Cantalapiedra et al., 2021) with the eggNOG v5.0 database (Huerta-Cepas et al., 2019) and Kyoto Encyclopedia of Genes and Genomes (KEGG) Orthology and Links Annotation (KOALA) (Kanehisa and Sato, 2020).

## 3 Results and discussion

### 3.1 Morphological, physiological, and biochemical characterization of strain FAM25158

**FIGURE 1:**
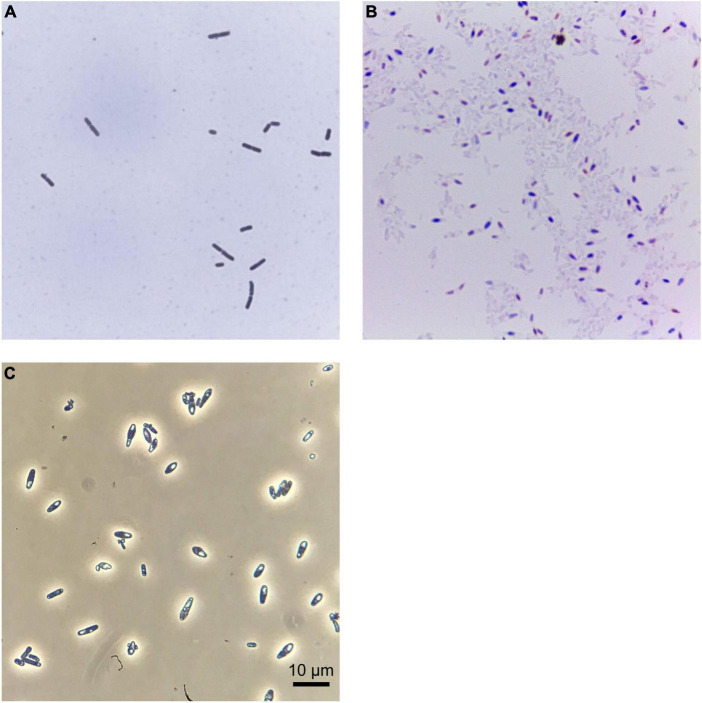
*Clostridium* sp. FAM25158 seen through different types of microscopy. Bright field optical microscopy of **(A)** Gram-stained cells and **(B)** spore-stained spore suspensions of strain FAM25158. **(C)** Phase contrast microscopy showing spores of strain FAM25158.

Standard microbiological tests identified strain FAM25158 as a Gram-negative, spore-forming, obligate anaerobic bacterium. When grown on RCM agar, strain FAM25158 produced circular, smooth, creamy-white, non-transparent colonies measuring 1.0–2.0 mm in width after 5 days at 37°C under anaerobic conditions. Cells were rod-shaped, approximately 1.2–1.6 μm wide, and variable in length, ranging from 3.0 to 8.0 μm, as determined by microscopy at 1,000 × magnification (Figure 1). The spores of FAM25158 were ellipsoidal and subterminal.

Microbial growth is strongly affected by intrinsic and extrinsic factors such as pH, salt concentration, and temperature. In the present study, the temperature range for growth of strain FAM25158 was found to be in the range of 20–40°C, while the reference *C. tyrobutyricum* strains showed growth between 20 and 45°C. As shown in Table 1, surprisingly, strain FAM25158 exhibited optimal growth at 30°C, a temperature notably lower than the optimal growth temperature observed for the reference *C. tyrobutyricum* strains (37°C) and the temperature previously reported in the literature for the species *C. tyrobutyricum* (Rainey et al., 2015; Yang et al., 2022). Furthermore, the maximum growth temperatures observed in our study were lower than the temperatures (43–50°C) reported in previous studies (Ruusunen et al., 2012; Yang et al., 2022). This observation highlighted the different temperature tolerance of the *Clostridium* strains. Moreover, strain FAM25158 showed the highest growth rate and shorter lag phase at 30°C; growth rates were about twofold higher at 30°C than at 37°C (data not shown). On the basis of the observed optimum temperature of strain FAM25158, we decided to include the temperature of 30°C for all further experiments testing pH and NaCl tolerance.

**TABLE 1:** Characteristics of *Clostridium* sp. strain FAM25158, *Clostridium tyrobutyricum* strains Cl_64, Cl_117, Cl_238, and DSM 2637^T^, *Clostridium pabulibutyricum* MJC39^T^, and *Clostridium algifaecis* MB9-7^T^.

Characteristics	*Clostridium* sp. FAM25158	*C. tyrobutyricum* Cl_64	*C. tyrobutyricum* Cl_117	*C. tyrobutyricum* Cl_238	*C. tyrobutyricum* DSM 2637^T^	*C. pabulibutyricum* MJC39^Ta^	*C. algifaecis* MB9-7^Tb^
Cell length (μm)	3.0–8.0	2.0–8.0	2.0–6.0	2.0–7.0	1.9–13.3	1.6–3.9	2.0–4.0
Cell width (μm)	1.2–1.6	1.0–1.5	1.0–1.5	1.0–1.6	1.1–1.6	0.4–0.8	0.2–0.3
Endospore location	Subterminal	Subterminal	Subterminal	Subterminal	Subterminal	Terminal	Subterminal
Temperature range (°C)	20–40 (optimum, 30°C)	20–42 (optimum, 37°C)	20–45 (optimum, 37°C)	20–45 (optimum, 37°C)	20–42 (optimum, 37°C)	20–42 (optimum, ND)	20–45 (optimum, 30–37°C)
pH range at 37°C	4.5–7.0	4.5–8.0	4.5–7.5	4.5–8.5	4.5–8.5	5.0–7.0	4.0–8.0
Tolerance to NaCl (%, w/v) at 37°C	0.5	2	1	2	2	2	2
**Acid formation from (API 20A):**							
Glucose	+	+	+	+	+	+	+
Mannitol	+	+	+	+	+	+	+
Lactose	−	−	−	−	−	+	−
Sucrose	+	−	−	−	−	−	−
Maltose	−	−	−	−	−	−	−
Salicin	−	−	−	−	−	−	−
Xylose	−	+	−	−	−	+	+
Arabinose	−	+	−	−	−	+	−
Glycerol	−	−	−	−	−	−	−
Cellobiose	−	−	−	−	−	−	−
Mannose	+	+	+	+	−	+	+
Melezitose	−	−	−	−	−	−	−
Raffinose	−	−	−	−	−	−	−
Sorbitol	−	−	−	−	−	+	−
Rhamnose	−	+	−	−	−	+	−
Trehalose	−	−	−	−	−	−	−
**Enzyme activities (API-ZYM):**							
Alkaline phosphatase	w	−	−	W	w	−	w
Esterase	w	+	w	W	w	−	−
Esterase Lipase	w	w	w	W	w	-	−
Leucine Arylamidase	+	+	−	+	+	+	+
Valine Arylamidase	w	w	−	W	−	−	+
Cystine Arylamidase	w	w	w	W	−	−	w
Acid Phosphatase	+	w	w	W	w	+	+
Naphthol-AS-BI-Phosphohydrolase	+	w	w	+	w	+	+

*^a^*Data taken from Kobayashi et al. (2017).

*^b^*Data taken from Wu et al. (2014). ND, not determined; +, positive; −, negative; w, weak.

At 37°C, the growth of FAM25158 was observed in a pH range of 4.5–7.0 (optimum, pH 6.0), which is somewhat narrower than the pH range observed for the reference strains of *C. tyrobutyricum* (4.5–8.5) (Table 1). At 30°C, however, strain FAM25158 showed growth in a pH range of 4.5–7.5 (optimum, pH 6.0) (detailed data now shown). The pH ranges of all reference *C. tyrobutyricum* strains were also within the pH range of 4.5–7.5 with an optimum of 6.0 at 30°C (detailed data now shown). Our findings are in agreement with previous studies reporting an optimum pH of 5.9–6.0 for *C. tyrobutyricum* (Wu and Yang, 2003; Zhu and Yang, 2004; Liu and Yang, 2006; Liu et al., 2006).

In the present study, the growth of strain FAM25158 was inhibited in the presence of >1.0% NaCl concentration. Similar results were reported by Podrzaj et al. (2020), who observed no activity of strain FAM25158 in an experimental cheese broth containing 1.0% NaCl at pH of 5.4 (incubation at 14, 20, and 37°C). Conversely, the reference *C. tyrobutyricum* strains showed tolerance to up to 2.0% NaCl. A previous *in vitro* study also reported that *C. tyrobutyricum* can grow at 2.0% NaCl, with only a few strains demonstrating tolerance to a higher NaCl concentration of 3.0% (Ruusunen et al., 2012). However, Bellassi et al. (2022) found that salt concentrations higher than 0.5% NaCl (w/w) were inhibitory for the *C. tyrobutyricum* strains tested. The inability of strain FAM25158 to grow in the presence of >1.0% NaCl could be attributed to the increased susceptibility of the microorganism to osmotic stress, which leads to desiccation of microbial cells and, to a greater extent, cell death and lysis (Davidson et al., 2012). Some studies have also shown that salt interferes with cellular enzymes, thus reducing the growth rate of bacteria (Gunde-Cimerman et al., 2018; Sun et al., 2022). Furthermore, previous studies have shown that high salinity inhibits germination of bacterial spores (Nagler et al., 2014; Nagler et al., 2016; Boix et al., 2022). For example, in a study by Nagler et al. (2014), some germination receptors of *Bacillus subtilis* spores were inhibited by a high NaCl concentration. In the context of this study, it is important to note that strain FAM25158 was originally isolated from Emmental cheese, which typically contains approximately 0.7–2.0% of added NaCl, corresponding to 2.0–5.0% water-phase NaCl (Guinee and Fox, 2017). Given this salt concentration in cheese, it is reasonable to hypothesize that the germination and subsequent outgrowth of FAM25158 spores may have been reduced or even inhibited by the presence of salt. Our findings suggest that strain FAM25158 might have been an adventitious contaminant in the cheese, rather than the sole agent responsible for the observed LBD.

However, it is worth considering that the inhibitory effect of NaCl on *Clostridium* sp. may vary depending on the presence of other factors that either act synergistically or are more important than NaCl alone (Garde et al., 2011b; Khanipour et al., 2016). According to Silvetti et al. (2018), a delay in the time until gas production of both spores and vegetative cells of *C. tyrobutyricum* and *C. sporogenes* was observed at temperatures lower than 20°C, and at 37°C, at pH levels of 5.75 or lower, with NaCl having no or little effect at concentrations up to 2.0%. However, the introduction of NaCl into food products typically enhances the inhibitory effect on clostridia.

To further identify phenotypic differences between strain FAM25158 and its closest *C. tyrobutyricum* relatives, we performed metabolic tests. Strain FAM25158 showed similar characteristics as the reference *C. tyrobutyricum* strains, as it was negative in the catalase and oxidase tests and was able to ferment glucose, mannitol, and mannose (Table 1). However, strain FAM25158 was distinguished from its closest phylogenetic relatives by its ability to ferment sucrose.

The enzymatic profile of strain FAM25158 showed some notable similarities with those of the reference *C. tyrobutyricum* strains (Table 1). Both strain FAM25158 and *C. tyrobutyricum* reference strains showed a shared absence of enzymatic activities of α-galactosidase, β-galactosidase, β-glucuronidase, α-glucosidase, β-glucosidase, N-acetyl-β-glucosaminidase, α-mannosidase, and α-fucosidase, which are likely to be involved in the degradation of polysaccharides for energy metabolism, as well as trypsin, and α-chymotrypsin (data now shown). Conversely, both strain FAM25158 and the reference strains showed activity of acid phosphatase, esterase, esterase lipase, and naphthol-AS-BI-phosphohydrolase. Furthermore, weak cystine arylamidase activity was observed in FAM25158 and all reference strains, except *C. tyrobutyricum* DSM 2637*^T^*, while leucine arylamidase activity was present in strain FAM25158 and all reference strains except *C. tyrobutyricum* Cl_117. Additionally, weak valine arylamidase activity was shared among FAM25158, *C. tyrobutyricum* Cl_64, and *C. tyrobutyricum* Cl_238. Weak alkaline phosphatase activity was also observed in FAM25158, *C. tyrobutyricum* Cl_238, and *C. tyrobutyricum* DSM 2637*^T^*, indicating a consistent characteristic within this subset of strains. It is known that both acid and alkaline phosphatases are usually present in raw milk and are produced by lactic acid bacteria during cheese ripening, contributing to the formation of cheese flavor (Magboul and McSweeney, 1999; Akuzawa and Fox, 2004). Similarly, the degradation of amino acids and lipids to short peptides and free fatty acids, respectively, also plays a role in determining cheese flavor (Herreros et al., 2003). We identified weak activities of some of these enzymes in strain FAM25158, their exact role in FAM25158 and their potential impact on cheese flavor development, however, remain unclear and require further investigation.

### 3.2 Strain FAM25158 belongs to the *Clostridium sensu stricto* cluster based on its chemotaxonomic profile

Chemotaxonomic characterization is one of the approaches used to assist systematists in the identification and classification of bacteria and archaea (Nouioui and Sangal, 2022). Therefore, we analyzed the fatty acid and polar lipid profiles of strain FAM25158 and compared it to its closely related type strains of the genus *Clostridium* (i.e., *Clostridium algifaecis* MB9-7*^T^* and *C. tyrobutyricum* DSM 2637*^T^*). In addition to *C. tyrobutyricum* DSM 2637*^T^*, three other *C. tyrobutyricum* strains (i.e., Cl_64, Cl_117, and Cl_238) were included in the polar lipid analysis as they showed the closest relationship to strain FAM25158.

The cellular fatty acid composition of strain FAM25158 revealed a spectrum of 13 fatty acids, with the major fatty acids identified as C_16:0_ FAME (59.9%), C_17:0_ *cyc* DMA (10.3%), C_16:1_ cis 9 DMA (9.1%), and C_16:1_ cis 9 FAME (7.4%) (Supplementary Table 2). The minor fatty acids were C_16:1_ cis 11 FAME (3.5%), C_18:0_ FAME (2.7%), C_14:0_ FAME (1.6%), C_16:0_ DMA (1.5%), C_14:0_ DMA (1.1%), C_17:0_ *cyc* cis 9 (1.1%), C_14:0_ aldehyde (0.7%), C_18:1_ cis 11 FAME (0.7%), and C_12:0_ FAME (0.3%). The major fatty acids of strain FAM25158 were similar to those of the reference strains, indicating that FAM25158 should be considered a member of *Clostridium sensu stricto.* However, noteworthy variation, such as the presence of C_12:0_ FAME, C_17:0_ *cyc* cis 9, and C_18:1_ cis 11 FAME, not observed in the reference strains suggest a potential distinctiveness of FAM25158 from close relatives.

The polar lipid profile of strain FAM25158 consisted of phosphatidylglycerol, eight aminolipids, and a few unidentified phospholipids, aminophospholipids, and lipids (Supplementary Figure 1), which was similar to the reference strains. This observation is consistent with those of other *Clostridium* species (Johnston and Goldfine, 1983; Guan and Goldfine, 2021). Overall, the results of the chemotaxonomic analysis showed that strain FAM25158 is closely related to members of *Clostridium sensu stricto*, yet the subtle variations observed underscore the need for further exploration and a more refined understanding of its taxonomic placement within the genus.

### 3.3 Taxonomic assignment of strain FAM25158 based on the 16S rRNA gene sequence and whole-genome based analysis

To provide a taxonomic assignment for strain FAM25158, we first used the 16S rRNA gene sequences of strain FAM25158 and all *Clostridium* type strains. The 16S rRNA gene of strain FAM25158 showed the highest sequence similarity (98.55%) to *C. tyrobutyricum* DSM 2637*^T^*, followed by *C. algifaecis* MB9-7*^T^* (96.14%), *Clostridium autoethanogenum* DSM 10061*^T^* (95.79%), *Clostridium ljungdahlii* DSM 13528*^T^* (95.67%), and *Clostridium pabulibutyricum* MJC39*^T^* (95.61%). However, the 16S rRNA gene sequence similarities for strain FAM25158 were lower than 94.70% with regard to other *Clostridium sensu stricto* type strains (Supplementary Table 3).

**FIGURE 2:**
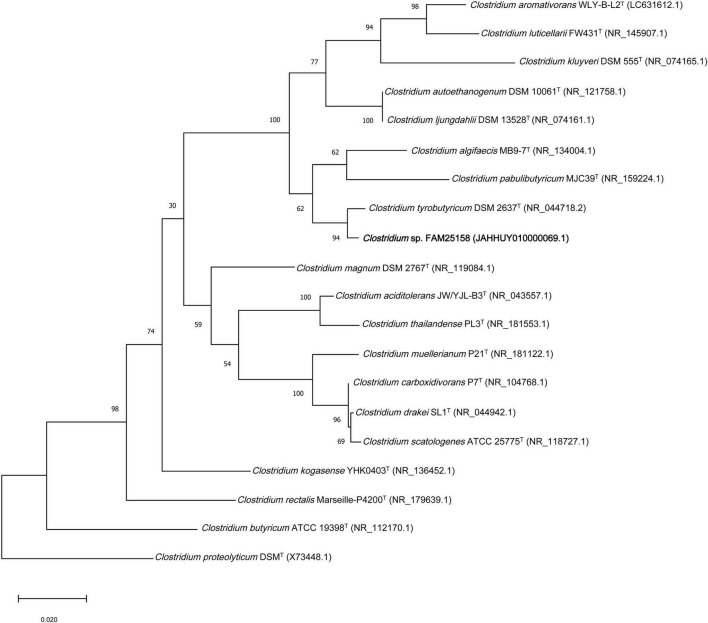
Maximum-likelihood tree constructed using the Kimura-2 parameter method showing the phylogenetic relationship of strain FAM25158, closely related type strains of the genus *Clostridium*, based on 16S rRNA gene sequence similarity. Bootstrap values (expressed as percentage of 1,000 replications) above 70% are shown at branch nodes. The sequence of *Clostridium proteolyticum* DSM 3090^T^ (Cluster II) was used as an outgroup. Bar, 0.02 substitutions per nucleotide position.

When a phylogenetic tree based on 16S rRNA gene sequences was built with the set of *Clostridium sensu stricto* type strains, a clear affiliation to the genus *Clostridium* could be seen (Figure 2). As expected, strain FAM25158 was grouped in a clade with *C. tyrobutyricum* DSM 2637^T^. This relationship was supported by high bootstrap values also found in the NJ tree (Supplementary Figure 2). Furthermore, in a 16S rRNA gene phylogenetic tree constructed with the three closely related *C. tyrobutyricum* strains, namely Cl_64, Cl_117, and Cl_238, strain FAM25158 was placed in a separate independent branch within the *C. tyrobutyricum* clade (Supplementary Figure 3). This observation showed that strain FAM25158 is genotypically distinct from *C. tyrobutyricum* strains, suggesting its potential classification to a different *Clostridium* species or subspecies within the *C. tyrobutyricum* species. However, it is important to emphasize the limitations of 16S rRNA gene sequence analysis in providing a definitive distinction among closely related species. While this method is the most commonly used for bacterial identification and phylogenetic analysis, its usefulness is limited because of the high percentage of sequence similarity between closely related species (Stackebrandt and Goebel, 1994). Several studies have demonstrated that the 16S rRNA gene phylogeny alone has limited ability to distinguish between clostridial species and strains (Collins et al., 1994; Bermudez et al., 2016; Yu et al., 2019; Podrzaj et al., 2022). To address this challenge, using a selection of a large and optimized set of genes or whole genome-based approaches can be valuable for the identification and characterization of certain prokaryote groups.

Therefore, to further define the taxonomic status of strain FAM25158, we computed the pairwise nucleotide-level comparisons between strain FAM25158 and the 2,768 *Clostridium* strains. We used two different bioinformatics methods, ANI and dDDH, as these approaches have been proposed to address the limitations associated with conventional laboratory-based DDH for evaluating the overall similarity of bacterial genomes (Colston et al., 2014). The ANI obtained after comparing the FAM25158 genome with other published *Clostridium* genomes revealed maximum ANIb values with 41 *C. tyrobutyricum* strains, ranging from 94.70 to 95.26%, which is just at the general threshold typically used for prokaryotic species delineation (95–96%) (Goris et al., 2007; Richter and Rossello-Mora, 2009; Kim et al., 2014; Jain et al., 2018). Indeed, the observed ANIb values were in the same range as the one that currently separates other *Clostridium* species such as *C. ljungdahlii* and *C. autoethanogenum* (94.44–99.15%). It is important to note, that better alignment improves the ANI values, which can be achieved by using complete genomes.

Although most bacterial species typically have ANI values ranging between 95 and 96% (Jain et al., 2018), it is worth noting that certain bacterial species can display higher intraspecies ANI values (Millán-Aguiñaga et al., 2017; Wright and Baum, 2018). Another suggested threshold for defining species boundaries is 96.5% ANI, which has proven to be closely mirroring a range of dDDH values, approximately 60–70% (Richter and Rossello-Mora, 2009; Wright and Baum, 2018). Indeed, the dDDH values between strain FAM25158 and the reference *C. tyrobutyricum* strains ranged between 60.2 and 62.5%. These values were below the recommended threshold for species delineation (70%) (Goris et al., 2007), indicating that strain FAM25158 is genetically distinct from the *C. tyrobutyricum* reference strains. Our analyses suggest that the natural boundaries between *Clostridium* species may vary. Given that the pairwise ANIb values between all other *C. tyrobutyricum* strains analyzed were higher than 98%, we consider that the compactness of *C. tyrobutyricum* could justify the classification of FAM25158 as a separate species because it is likely that FAM25158 harbors a large set of genetic traits that may not be present in other *C. tyrobutyricum* strains. However, given the fact that FAM25158 is a single representative strain, clear conclusions cannot be drawn based on the current data. Taxonomic classification of this strain as a separate species requires an expended dataset incorporating additional strains exhibiting similar traits (Felis and Dellaglio, 2007). Such dataset would facilitate a robust taxonomic assignment and contribute to a more comprehensive understanding of genetic diversity within the *Clostridium* group.

### 3.4 General genomic features and functional annotation of strain FAM25158

The genome of FAM25158 consisted of a singular circular chromosome and had a total sequence length of 3.09 Mbp within 152 contigs. Furthermore, the average GC-content of the FAM25158 was 30.60 mol%, which is within the range of GC-content (28.0–32.0 mol%) reported for the other members of the *Clostridium sensu stricto* cluster (Udaondo et al., 2017). Genome annotation allowed the prediction of 2,778 protein-coding sequences, 42 tRNA-coding genes, and two rRNA-coding genes (Table 2).

**TABLE 2:** Genome characteristics of the bacterial strain FAM25158 and *Clostridium tyrobutyricum* strains Cl_64, Cl_117, Cl_238, and DSM 2637^T^.

Bacterial strain	GenBank assembly accession number	Genome size (Mbp)	GC-content (mol%)	Contigs	Coding sequences (CDS)	Genes	rRNA	tRNA
*Clostridium* sp. FAM25158	GCA_019144085.1	3.09	30.60	152	2,778	3,037	2	42
*C. tyrobutyricum* Cl_64	GCA_019144065.1	3.17	31.00	110	3,017	3,228	4	46
*C. tyrobutyricum* Cl_117	GCA_019143985.1	3.11	31.10	117	2,958	3,172	4	46
*C. tyrobutyricum* Cl_238	GCA_019143995.1	3.32	30.80	107	3,132	3,362	4	48
*C. tyrobutyricum* DSM 2637^T^	GCA_000429805.1	3.13	31.00	44	3,018	3,157	19	63

The analysis of Clusters of COGs of proteins revealed a total of 2,731 protein-coding genes to be present in the FAM25158 genome, and 2,207 (80,81%) protein-coding sequences were classified as functional genes, excluding those classified as functionally unknown. The majority of predicted genes in strain FAM25158, as expected, were involved in COG categories related to basic cellular functions, such as “amino acid transport and metabolism” (237, 10.74%), “transcription” (224, 10.15%), “energy production and conversion” (221, 10.01%), and “replication, recombination, and repair” (183, 8.29%) (Supplementary Figure 4). This coincides with a recent study that found that approximately 24.64% of the genes present in all *Clostridium sensu stricto* tested genomes were predicted to function in metabolism including amino acid transport and metabolism, with the most frequent portion (Udaondo et al., 2017).

### 3.5 Comparative genomic analyses

To complement our previous analyses and to highlight the unique features of strain FAM25158, a pan-genomic analysis was carried out among strain FAM25158 and the four reference *C. tyrobutyricum* strains (i.e., Cl_64, Cl_117, Cl_238, and DSM 2637^T^). The five strains shared 1,513 genes (i.e., core genes), while an additional 10,103 genes were present in at least one but not all of the strains (i.e., accessory genes). Among these genes, 289 to 1,195 genes were exclusive to each genome (i.e., unique genes). Notably, strain FAM25158 stood out with the largest number of unique genes (1,195), indicating that it may possess unique features compared to the four *C. tyrobutyricum* strains. Of the 1,195 unique genes identified in FAM25158, more than half (55.48%) were classified as hypothetical proteins with no functional prediction. This observation suggests that considerable effort will be required to unravel the putative functions of these unique genes.

Furthermore, a comparison of the distribution of unique genes to COG functional categories revealed differences between strain FAM25158 and the four reference *C. tyrobutyricum* strains (Supplementary Figure 5). For instance, categories “intracellular trafficking and secretion,” “posttranslational modification, protein turnover, and chaperones,” and “translation, ribosomal structure, and biogenesis” were more abundant in strain FAM25158 compared to the reference *C. tyrobutyricum* strains. Specifically, genes encoding chaperones required for protein folding (i.e., *dnaJ*, *grpE*, *hslO*, and *prsA*), cold and heat shock proteins (i.e., *cspA*, *cplA*, and *hsp*), and genes encoding for secretion system enzymes involved in conjugation (i.e., *comEA*, *comEC*, *comFB*, *flgB*, *fliHNQ*, *secA*, *xpsE*, and *epsF*) were identified in FAM25158 but not in *C. tyrobutyricum* strains. These genes are associated with fundamental metabolic processes, and many of them have been found to play a role in enabling bacteria to adapt to challenging environmental conditions, such as heat, cold and acid stress. However, it is interesting to note that several cold shock proteins (Csps) and the high temperature requirement B (HtrB)-like protease were absent in strain FAM25158 but present in the reference *C. tyrobutyricum* strains. The Csps are known to contribute to cold and osmotic stress tolerance in various organisms including *C. botulinum*, *C. acetobutylicum*, *Listeria monocytogenes*, and *Bacillus subtilis* (Bahl et al., 1995; Söderholm et al., 2011). In addition, the HtrA- and HtrB-like proteases have been shown to play a role in protein homeostasis and quality control in both Gram-positive and -negative bacteria (Chatfield et al., 1992; Ibrahim et al., 2004; Chitlaru et al., 2011; Bakker et al., 2014). The presence of such genes in *C. tyrobutyricum* suggests the possibility that the gene products may represent the main cellular response to increased osmolarity, and therefore their presence may be essential in environments with high salt concentrations. This finding is supported by the fact that strain FAM25158 showed reduced growth in the presence of NaCl. However, further mutation or proteomic studies may be required to determine the exact role of these genes in FAM25158 and *C. tyrobutyricum*.

Moreover, the COG categories related to “nucleotide transport and metabolism” and “energy production and conversion” were also more enriched in the unique genes of FAM25158 compared to *C. tyrobutyricum*. Specifically, some of the genes belonging to these categories are involved in proton exporting functions, such as ATPase, and the overall electron transferase chain proteins, such as pyruvate:ferrodoxin oxidoreductase. The higher abundance of these gene categories suggests increased intracellular survival, since they might efficiently utilize the nutrients present in the environment to generate energy.

Another significant group of unique genes of FAM25158 were those involved in the “lipid transport and metabolism” category, such as the genes encoding for acetyl-CoA synthetase (*pls*), genes encoding fatty acid synthase (*fabD* and *fabG*), and genes encoding for phospholipid acetyl transferases. This observation implies that the products of these genes could potentially impact the lipid composition of the cell membrane. Previous studies have shown that bacteria can adapt to low pH environments by preferentially using acetyl-CoA for the biosynthesis of saturated fatty acids instead of isoprenoids to stabilize the cell membrane (Fernandez et al., 2008). Hence, it would be interesting to further investigate these genes by gene expression analysis and mutant characterization to further understand the genetic mechanisms involved in *Clostridium* survival.

There were fewer unique genes involved in the “replication, recombination, and repair” and “transcription” categories in strain FAM25158 compared to the reference *C. tyrobutyricum* strains, which suggests that these genes are critical for the survival of *C. tyrobutyricum* or could possibly be attributed to their ability to adapt to environmental challenges. However, further exploration is needed to identify the exact role of such genes.

Overall, the functional gene differences between strain FAM25158 and the reference *C. tyrobutyricum* strains may be involved in the adaptation to environmental changes and could also discriminate the strain FAM25158 from other *C. tyrobutyricum* strains at the genome level. The fact that approximately 24.01% of the unique genes of FAM25158 were not assigned to any COG function suggests that orthologs for these genes are not present in any of the species studied. Their analyses may provide clues about other functions and novel mechanisms in strain FAM25158.

### 3.6 *In silico* analysis revealed the presence of genes associated to carbohydrate metabolism in FAM25158

Butyric acid bacteria are known to occupy various environments, requiring different sets of metabolic capabilities necessary for successful habitat colonization. *In silico* genome analysis revealed that strain FAM25158 possessed genes predicted to be involved in the metabolism of glucose, mannitol, mannose, and sucrose. These data are in agreement with the phenotypic characterization and with the notion that the species *C. tyrobutyricum* (closely related to FAM25158) can ferment simple carbohydrates (Ivy and Wiedmann, 2014).

We then compared the identified genes in the FAM25158 genome with the genetic content of the four reference *C. tyrobutyricum* strains. In general, the genomes of the analyzed strains revealed the presence of genes involved in the metabolic pathway for butyric acid fermentation, indicating the conserved capacity of these bacteria to ferment butyric acid. Specifically, the analyzed genomes possessed genes encoding enzymes required for the conversion of glucose to pyruvate in the Embden-Mayerhof-Parnas pathway, such as pyruvate:ferredoxin oxidoreductase (*pfor*) that converts pyruvate into acetyl-CoA, and fructose-1,6-biphosphatase (*fbp*), acetate kinase (*ack*), lactate racemase (*larA*), L-lactate dehydrogenase (*ldh*), and hydroxyacylglutathione hydrolase (*gloB*) that are involved in the synthesis of L-lactate from pyruvate. Furthermore, genes involved in butyric acid fermentation were also found in all analyzed genomes, and included phosphotransacetylase (*pta*) that catalyzes the conversion of acetyl-CoA into acetic and butyric acid, acetate kinase (*ack*) for catalytic conversion of acetyl-CoA to acetic acid, acetyl-CoA acetyltransferase (*atoB*), 3-hydroxybutyryl-CoA dehydrogenase (*hbd*), and butyryl-CoA dehydrogenase (*bcd*) that convert acetyl-CoA to butytyl-CoA, as well as butyryl-CoA/acetate CoA transferase (*cat2*) that converts butylyl-CoA to butyric acid.

Additionally, the presence of genes encoding for the transport proteins and enzymes involved in the metabolism of mannose and mannitol via the phosphoenolpyruvate-dependent sugar phosphotransferase system (PTS) was also identified in the genomes of FAM25158 and the four reference *C. tyrobutyricum* strains. Specifically, the genes encoding mannose-6-phosphate isomerase (*manA*), phosphomannomutase (*algC*) that catalyzes the transfer of phosphate from D-mannose-1-phosphate to D-mannose-6-phosphate, a precursor of fructose-6-phosphate, and several genes encoding mannose-specific EIIABCC components were found in all genomes analyzed. Thus, these genes may be responsible for mannose utilization. We also found that the genomes of FAM25158 and the four reference *C. tyrobutyricum* strains contain the *mtl* operon comprising four genes, namely the gene *mtlA* encoding the D-mannitol-specific enzyme II transporter (EIICAB), followed by *mtlR* encoding a putative transcriptional regulator, *mtlF* encoding the EIIA domain of the phosphotransferase, and the gene *mtlD* encoding mannitol-1-phosphate 5-dehydrogenase. These genes are involved in the transport of mannitol. This observation suggests, that the gene products of this cluster may be responsible for mannitol utilization.

Despite similarities, genes involved in the transport and hydrolysis of sucrose, and the subsequent phosphorylation of the resulting fructose, were found in strain FAM25158, but not in the genomes of the reference *C. tyrobutyricum* strains. These genes included sucrose-6-phosphate hydrolase (*scrB*), which cleaves sucrose-6-phosphate to yield glucose-6-phosphate and fructose and is associated with the PTS, a fructokinase (*scrK*) involved in the catalysis of fructose to fructose-6-phosphate, and a gene encoding for the EIIBCA/ EIIBC component of the sucrose PTS system (*scrA*). We observed that these genes were located next to each other, forming a gene cluster of 4,373 bp in length (including the intergenic sequences). Similarly, the presence of the *scrARBK* cluster has also been detected in *C. beijerinckii*, *C. acetobutylicum*, and *Clostridium perfringens* (Reid et al., 1999; Nolling et al., 2001; Shimizu et al., 2002). These data suggest that the *src* genes identified in FAM25158 may be responsible for the sucrose utilization. Thus, these genes may be important targets for future studies to determine their exact role in carbohydrate utilization in FAM25158.

## 4 Conclusion

In the present study, a *Clostridium* strain FAM25158 isolated from Emmental cheese was characterized by phenotypic, chemotaxonomic, and genomic analyses, and compared to a set of closely related *C. tyrobutyricum* strains. Using WGS-based analyses, we showed that strain FAM25158 does not completely resemble *C. tyrobutyricum*, but differs in several important aspects from *C. tyrobutyricum* genomes. The detailed comparative genomic analyses performed using strain FAM25158 together with *C. tyrobutyricum* Cl_64, *C. tyrobutyricum* Cl_117, *C. tyrobutyricum* Cl_238, and *C. tyrobutyricum* DSM 2637^T^ allowed us to gain unique insights into the genetics and metabolic potential of FAM25158, suggesting its possible utilization of sucrose, which was consistent with the observed ability to ferment sucrose during biochemical characterization. In addition, genomic analysis also revealed the absence of genes encoding for cold shock proteins and genes involved in osmolality in the genome of FAM25158. This finding corroborates with the unique phenotypic characteristics of strain FAM25158, which showed a lower optimum temperature for growth than its closest relatives of the species *C. tyrobutyricum*, and no tolerance to salt concentrations higher than 1%. However, a high percentage of genes with unknown functions indicates that many unique features of strain FAM25158 may not yet be reported. While our investigation highlights the unique genotypic and phenotypic characteristics of strain FAM25158, the absence of clear species delineation thresholds in genomic analyses necessitates caution in definitively designating it as a separate species. Therefore, further studies and more strains related to FAM25158 would be needed to resolve its exact taxonomic classification. However, our results represent a remarkable contribution to the understanding of the diversity and characteristics of *Clostridium* strains associated with cheese spoilage.

## Data availability statement

The original contributions presented in the study are included in the article/Supplementary material, further inquiries can be directed to the corresponding author.

## Author contributions

LP: Conceptualization, Data curation, Formal analysis, Investigation, Visualization, Writing – original draft, Writing – review and editing. JB: Conceptualization, Project administration, Supervision, Writing – review and editing. PS: Investigation, Writing – review and editing. TK: Investigation, Writing – review and editing. KD: Conceptualization, Project administration, Supervision, Writing – review and editing.

## Footnotes

1
https://www.bacterio.net/

2
https://www.ncbi.nlm.nih.gov/genbank/

3
https://www.ezbiocloud.net/

4
https://blast.ncbi.nlm.nih.gov/Blast.cgi

5
https://www.ncbi.nlm.nih.gov/refseq/

6
https://github.com/widdowquinn/pyani

7
https://ggdc.dsmz.de/ggdc.php

8
https://tygs.dsmz.de/
