# Supplementary material for: Clostridium strain FAM25158, a unique endospore-forming bacterium related to Clostridium tyrobutyricum and isolated from Emmental cheese shows low tolerance to salt
Source: Front Microbiol. 2024 Feb 13;15:1353321. doi: 10.3389/fmicb.2024.1353321 (PMC10897056; doi:10.3389/fmicb.2024.1353321)
Supplement: Supplementary file 1 [file Table_1.DOCX]

**Supplementary Table 1:** List of valid type strains of *Clostridium* spp. used in phylogenetic construction based on 16S rRNA gene sequences.

| Strain | Accession | Reference |
| --- | --- | --- |
| *Clostridium aciditolerans* JW/YJL-B3^T^ | NR_043557 | Lee et al. (2007) |
| *Clostridium algifaecis* MB9-7^T^ | NR_134004 | Wu et al. (2014) |
| *Clostridium aromativorans* WLY-B-L2^T^ | LC631612 | Luo et al. (2023) |
| *Clostridium autoethanogenum* DSM 10061^T^ | NR_121758 | Brown et al. (2014) |
| *Clostridium beijerinckii* JCM 1390^T^ | NR_113388 | Unpublished |
| *Clostridium butyricum* ATCC 19398^T^ | NR_112170 | Kikuchi et al. (2002) |
| *Clostridium drakei* SL1^T^ | NR_044942 | Liou et al. (2005) |
| *Clostridium kluyveri* DSM 555^T^ | NR_074165 | Seedorf et al. (2008) |
| *Clostridium ljungdahlii* DSM 13528^T^ | NR_074161 | Köpke et al. (2010) |
| *Clostridium luticellarii* FW431^T^ | NR_145907 | Wang et al. (2015) |
| *Clostridium pabulibutyricum* MJC39^T^ | NR_159224 | Kobayashi et al. (2017) |
| *Clostridium proteolyticum* DSM 3090^T^ | X73448.1 | Unpublished |
| *Clostridium scatologenes* ATCC 25775^T^ | NR_118727 | Liou et al. (2005) |
| *Clostridium sporogenes* JCM 1416^T^ | NR_113245 | Unpublished |
| *Clostridium tyrobutyricum* DSM 2637^T^ | NR_044718 | Unpublished |
| *Clostridium thaliandense* PL3^T^ | NR_181553 | Chaikitkaew et al. (2022) |
| *Clostridium muellerianum* P21^T^ | NR_181122 | Doyle et al. (2022) |

Brown, S.D., Nagaraju, S., Utturkar, S., De Tissera, S., Segovia, S., Mitchell, W., et al. (2014). Comparison of single-molecule sequencing and hybrid approaches for finishing the genome of *Clostridium autoethanogenum* and analysis of CRISPR systems in industrial relevant Clostridia. *Biotechnol Biofuels* 7**,** 40. doi: 10.1186/1754-6834-7-40.

Chaikitkaew, S., In-chan, S., Singkhala, A., Tukanghan, W., Mamimin, C., Reungsang, A., et al. (2022). *Clostridium thailandense* sp. nov., a novel CO2-reducing acetogenic bacterium isolated from peatland soil. *Int J Syst Evol Microbiol* 72(2). doi: 10.1099/ijsem.0.005254.

Doyle, D.A., Smith, P.R., Lawson, P.A., and Tanner, R.S. (2022). *Clostridium muellerianum* sp. nov., a carbon monoxide-oxidizing acetogen isolated from old hay. *Int J Syst Evol Microbiol* 72(3). doi: 10.1099/ijsem.0.005297.

Kikuchi, E., Miyamoto, Y., Narushima, S., and Itoh, K. (2002). Design of species-specific primers to identify 13 species of *Clostridium harbored* in human intestinal tracts. *Microbiol Immunol* 46(5)**,** 353-358. doi: 10.1111/j.1348-0421.2002.tb02706.x.

Kobayashi, H., Nakasato, T., Sakamoto, M., Ohtani, Y., Terada, F., Sakai, K., et al. (2017). *Clostridium pabulibutyricum* sp. nov., a butyric-acid-producing organism isolated from high-moisture grass silage. *Int J Syst Evol Microbiol* 67(12)**,** 4974-4978. doi: 10.1099/ijsem.0.002387.

Köpke, M., Held, C., Hujer, S., Liesegang, H., Wiezer, A., Wollherr, A., et al. (2010). *Clostridium ljungdahlii* represents a microbial production platform based on syngas. *Proc Natl Acad Sci U S A* 107(29)**,** 13087-13092. doi: 10.1073/pnas.1004716107.

Lee, Y.-J., Romanek, C.S., and Wiegel, J. (2007). *Clostridium aciditolerans* sp. nov., an acid-tolerant spore-forming anaerobic bacterium from constructed wetland sediment. *Int J Syst Evol Microbiol* 57(2)**,** 311-315. doi: 10.1099/ijs.0.64583-0.

Liou, J.S., Balkwill, D.L., Drake, G.R., and Tanner, R.S. (2005). *Clostridium carboxidivorans* sp. nov., a solvent-producing clostridium isolated from an agricultural settling lagoon, and reclassification of the acetogen *Clostridium scatologenes* strain SL1 as *Clostridium drakei* sp. nov. *Int J Syst Evol Microbiol* 55(Pt 5)**,** 2085-2091. doi: 10.1099/ijs.0.63482-0.

Luo, Q., Zheng, J., Zhao, D., and Liu, D. (2023). *Clostridium aromativorans* sp. nov., isolated from pit mud used for producing Wuliangye baijiu. *Antonie Van Leeuwenhoek* 116(7)**,** 739-748. doi: 10.1007/s10482-023-01841-0.

Seedorf, H., Fricke, W.F., Veith, B., Brüggemann, H., Liesegang, H., Strittmatter, A., et al. (2008). The genome of *Clostridium kluyveri*, a strict anaerobe with unique metabolic features. *Proc Natl Acad Sci U S A* 105(6)**,** 2128-2133. doi: 10.1073/pnas.0711093105.

Wang, Q., Wang, C.D., Li, C.H., Li, J.G., Chen, Q., and Li, Y.Z. (2015). *Clostridium luticellarii* sp. nov., isolated from a mud cellar used for producing strong aromatic liquors. *Int J Syst Evol Microbiol* 65(12)**,** 4730-4733. doi: 10.1099/ijsem.0.000641.

Wu, Y.F., Zheng, H., Wu, Q.L., Yang, H., and Liu, S.J. (2014). *Clostridium algifaecis* sp. nov., an anaerobic bacterial species from decomposing algal scum. *Int J Syst Evol Microbiol* 64(Pt 11)**,** 3844-3848. doi: 10.1099/ijs.0.064345-0.
