# Supplementary material for: Clostridium strain FAM25158, a unique endospore-forming bacterium related to Clostridium tyrobutyricum and isolated from Emmental cheese shows low tolerance to salt
Source: Front Microbiol. 2024 Feb 13;15:1353321. doi: 10.3389/fmicb.2024.1353321 (PMC10897056; doi:10.3389/fmicb.2024.1353321)
Supplement: Supplementary file 4 [file Data_Sheet_1.docx]

Supplementary Material

*Clostridium* strain FAM25158, a unique endospore-forming bacterium related to *Clostridium tyrobutyricum* and isolated from Emmental cheese shows low tolerance to salt

Lucija Prinčič^1^, Johanna Burtscher^1^*, Paul Sacken^1^, Tina Krajnc^2^, Konrad J. Domig^1^

^1^Department of Food Science and Technology, Institute of Food Science, University of Natural Resources and Life Sciences, Vienna, Vienna, Austria

^2^Biotechnical Faculty, University of Ljubljana, Ljubljana, Slovenia

*** Correspondence:**Johanna Burtscher
[johanna.burtscher@boku.ac.at](mailto:johanna.burtscher@boku.ac.at)


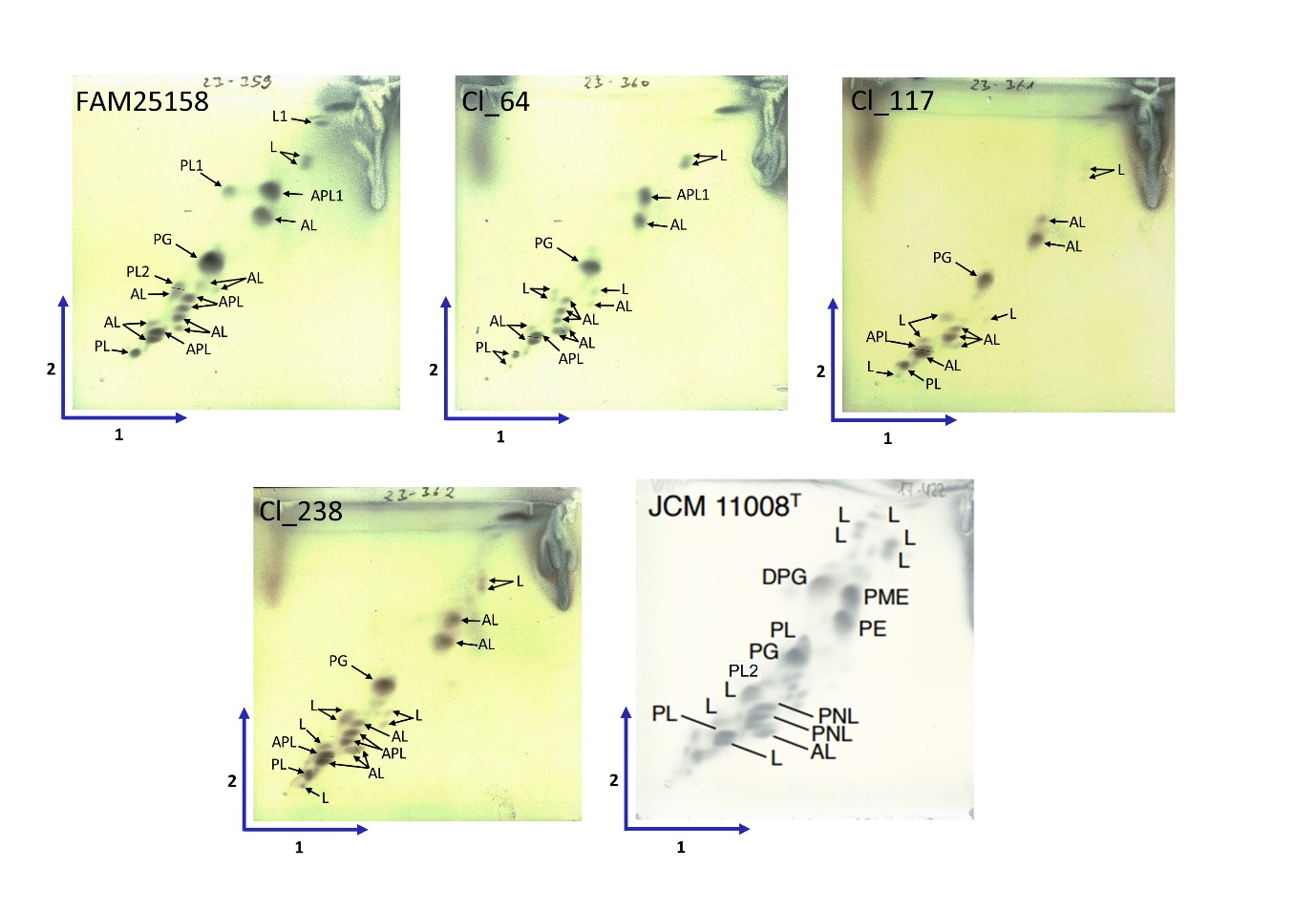


**Supplementary Figure 1.** Polar lipid profiles of strain FAM25158 and the close relatives of *Clostridium tyrobutyricum* after separation by two-dimensional thin-layer chromatograms. APL (PNL), aminophospholipid; AL, aminolipid; DPG, diphosphatidylglycerol; PE, phosphatidylethanolamine; PME, phosphatidylmethylethanolamine; PL, phospholipid; PG, phosphatidylglycerol; L, unidentified lipid.


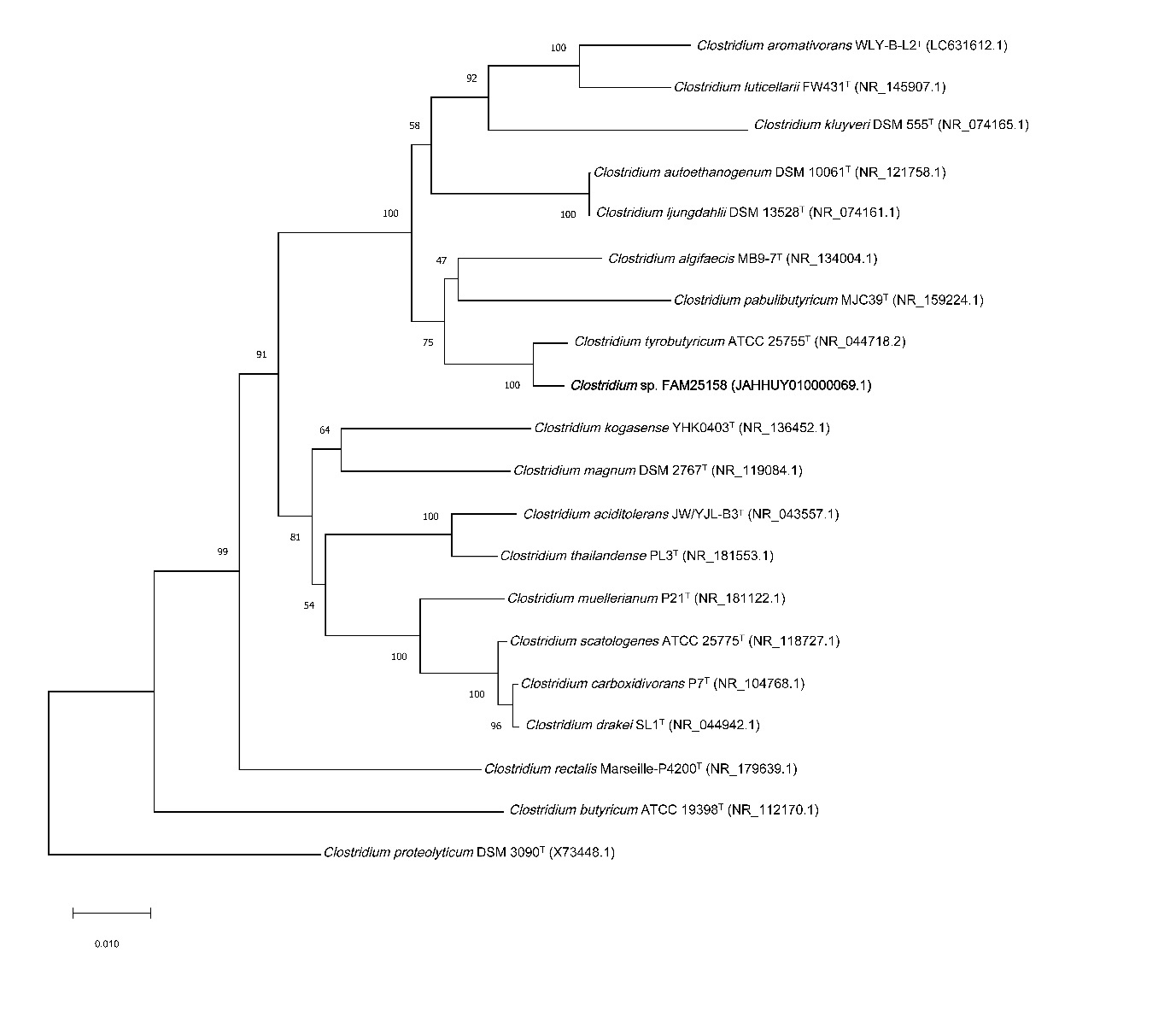


**Supplementary Figure 2:** Neighbor-joining tree showing the phylogenetic relationship of strain FAM25158 and other closely related type strains of *Clostridium* based on 16S rRNA gene sequences similarity. The sequence of *Clostridium proteolyticum* DSM 3090^T^ (Cluster II) was used as the outgroup. Bar, 0.01 substitutions per nucleotide position.

**
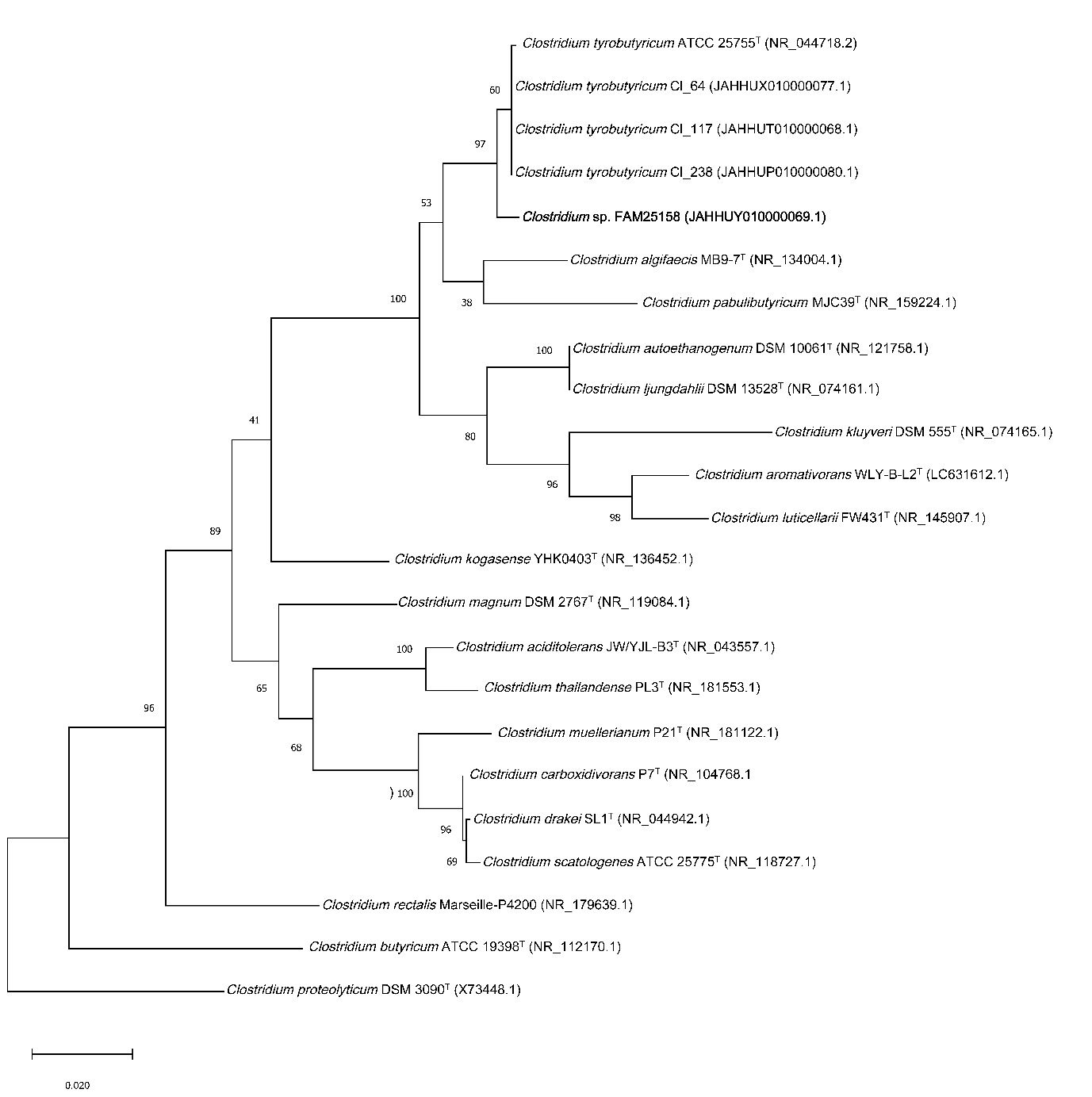
**

**Supplementary Figure 3:** Maximum-likelihood tree constructed using Kimura-2 parameter method showing the phylogenetic relationship of strain FAM25158, closely related type strains of *Clostridium* and *Clostridium tyrobutyricum* strains Cl_64, Cl_117, and Cl_238 based on 16S rRNA gene sequence similarity. Bootstrap values (expressed as percentage of 1,000 replications) above 70% are shown at branch nodes. The sequence of *Clostridium proteolyticum* DSM 3090^T^ (Cluster II) was used as the outgroup. Bar, 0.02 substitutions per nucleotide position.

**
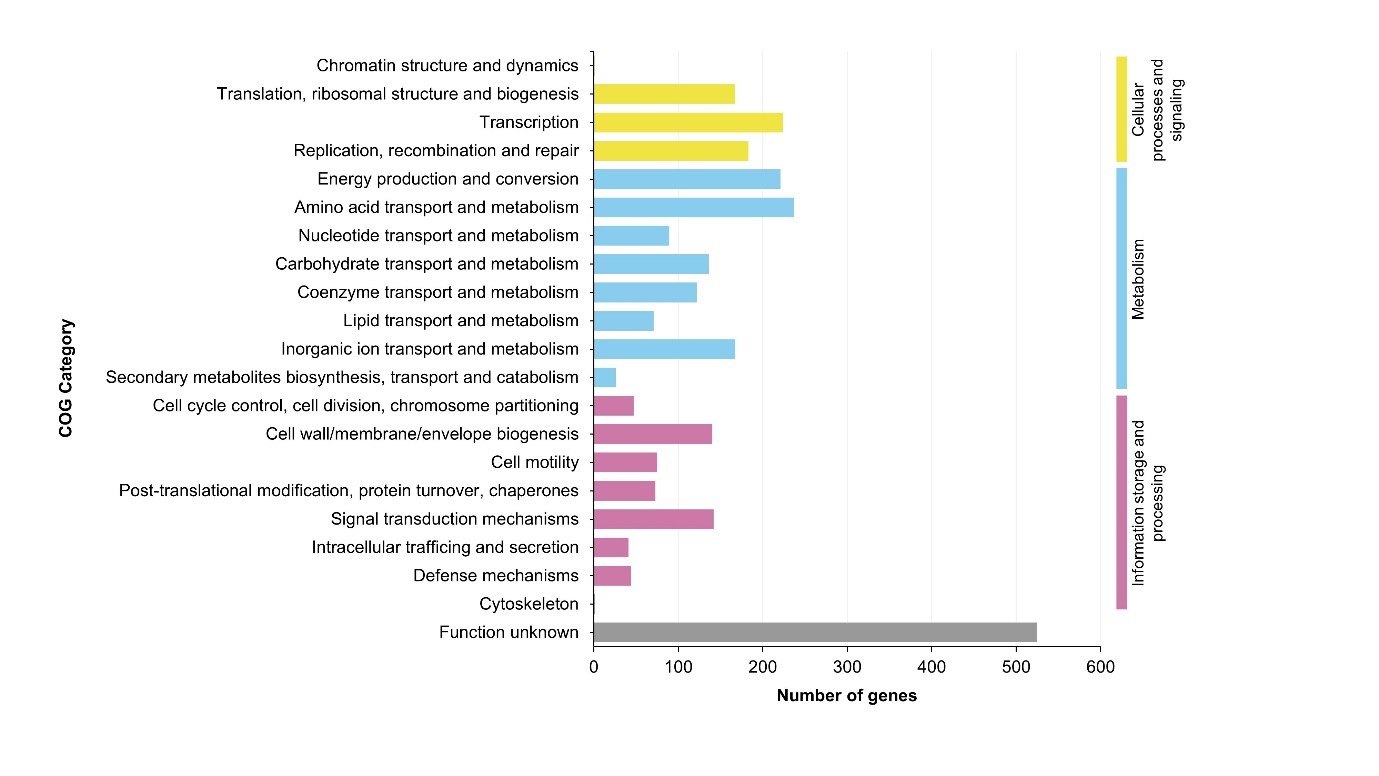
**

**Supplementary Figure 4:** Distribution of functional Clusters of Orthologous Groups (COGs) in the genome of FAM25158.


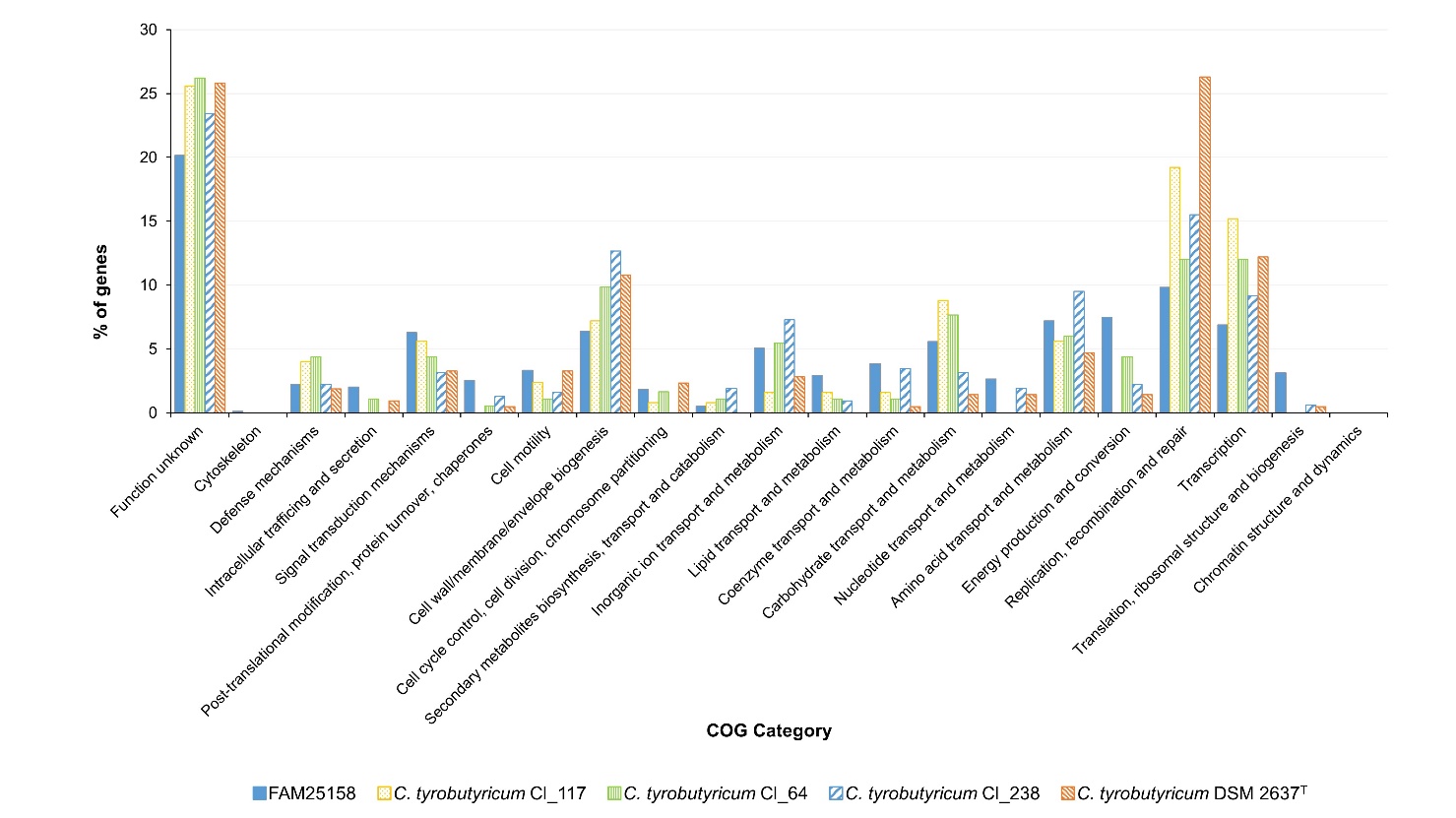


**Supplementary Figure 5:** Functional characterization of unique genes of the FAM25158 strain and *Clostridium tyrobutyricum* strains Cl_64, Cl_117, Cl_238, and DSM 2637^T^ according to the Clusters of Orthologous Groups (COGs) database.
